# LDL and IL-6 induce TGF-β1 release and mast cell migration toward intimal macrophages

**DOI:** 10.21203/rs.3.rs-3218621/v1

**Published:** 2023-08-04

**Authors:** Heng Yu, Radhika R. Josi, Ankur Khanna, Damir B. Khismatullin

**Affiliations:** Tulane University; Tulane University; Tulane University; Tulane University

**Keywords:** atherogenesis, macrophage, mast cell, TGF-β1, IL-6, LDL

## Abstract

**Objective and design:**

This study tests the hypothesis that mast cells migration to the artery’s intimal layer occurs due to release of TGF-β1 from macrophages exposed to LDL and IL-6.

**Material or subjects:**

Human monocytic cells (THP-1), human mast cells (LUVA), and human umbilical vein endothelial cells (HUVEC).

**Treatment:**

THP-1 cells were differentiated into M0, M1, and M2 macrophages, which were then treated with LDL, oxidized LDL (oxLDL), IL-6, or a combination of LDL and IL-6. LUVA cells and HUVEC were exposed to conditioned media from untreated and treated macrophages. LUVA cells were also exposed to TGF-β1.

**Methods:**

The concentration of TNF-α and TGF-β1 released from macrophages was measured by ELISA. The migration of LUVA cells in a microfluidic channel was assessed for 12 h. THP-1 cell adhesion to HUVEC was investigated under static conditions.

**Results:**

Inflammatory (M1) macrophages exposed to LDL + IL-6 or oxLDL released TGF-β1 at the level close to anti-inflammatory (M2) macrophages. These M2-like cells kept their inflammatory properties, based on adhesion data. The LUVA cells migrated to TGF-β1 or the conditioned medium from M2-like macrophages.

**Conclusions:**

LDL in combination with IL-6 repolarized macrophages from M1 to M2-like cells, which attracted mast cells via TGF-β1.

## Introduction

The development of atherosclerotic plaques in the artery wall is driven by abnormal communication between blood cells, endothelial cells, and tissue-resident cells in a presence of inflammatory mediators and low-density lipoproteins. This process, referred to as atherogenesis, starts with adhesion of blood monocytes to activated vascular endothelium, which causes subendothelial migration of these cells and their differentiation into macrophages[1]. Monocyte extravasation to the tunica intima is regulated by endothelial cell adhesion molecules [2–4] and monocyte chemokines [5]. With the help of other tissue-resident cells, cytokines, and oxidized low-density lipoproteins (oxLDL), macrophages located in the intimal layer of the artery transform into lipid-rich foam cells, thereby causing the formation of a fatty streak, the first clinical sign of atherosclerosis [6].

The tissue-resident macrophages can be in a resting state (M0 type) or be polarized into pro-inflammatory (M1 type) or anti-inflammatory (M2 type) macrophages, depending on the stimuli present [7]. In early stages of atherosclerosis, most macrophages are M1 type [8] because IFN-γ, the major cytokine for pro-inflammatory polarization of macrophages, is more abundant than IL-4, the cytokine that induces anti-inflammatory polarization [9]. M1 macrophages initiate the immune response by releasing TNF-α and other pro-inflammatory mediators. They also produce nitric oxide or reactive oxygen intermediates (ROI) to protect against bacterial and viral pathogens. M2 macrophages assist in tissue repair [10] by inducing cell proliferation and collagen production via transforming growth factor beta 1 (TGF-β1) release [11]. Supporting the proper balance between M1 and M2 types is essential for immunity and tissue repair, but it does not occur during atherogenesis. In this pathophysiological process, when M1 macrophages switch to M2 type, they remain in this state and eventually become foam cells [12].

It is still unclear which factors interfere with macrophage polarization. However, recent experimental evidence points out that low-density lipoproteins, mast cells, and chemicals released during mast cell degranulation may be involved [13, 14]. Initially located in the adventitial layer of the artery wall, mast cells migrate to and become co-localized with macrophages in the intimal layer [15]. They contribute to the conversion of M2 macrophages to foam cells [16] and promote the atherosclerotic lesion formation through histamine release [17]. During late stages of atherosclerosis, they are located, together with macrophages, in the shoulder regions of vulnerable plaques [18]. The mechanism of how mast cells become co-localized with intimal macrophages is not yet explored.

Mast cells would migrate to the intima in response to chemotactic stimuli released by the cells present in this region. One of the chemokines involved in this process could be TGF- β1, which is known to induce the direct migration of mouse mast cells to the sites of bacterial infection at femtomolar concentrations [19, 20] through binding its serine/threonine receptors [21]. TGF- β1 can be produced by intimal M2 macrophages through SMAD2 signaling [17], but this requires re-polarization of M1 macrophages, predominantly present in early atherosclerotic sites, into M2 type. Cytokines such as interleukin-6 (IL-6) and/or lipoproteins can be involved in the process of M1 to M2 transformation. IL-6 increases the expression of CD206, a M2 macrophage marker, and the CD206/CD86 expression ratio, where CD86 is a M1 macrophage marker [22]. Macrophages are also sensitive to low-density lipoprotein (LDL), especially to its oxidized form, oxLDL. For example, LDL upregulates the expression of integrins on monocytes and accelerates their transformation into M0 macrophages [23], while oxLDL enhances cytokine (TNF-α) release from macrophages [13], and, at the same time, assists in the transformation of M1 macrophages into foam cells [24]. Clinical data support important roles of IL-6 and oxLDL in atherosclerosis and coronary artery disease. Particularly, patients with family history of premature coronary artery disease have a significantly higher level of plasma IL-6 [25]. There is also a positive correlation between the plasma level of oxLDL and coronary artery disease [26].

Based on these data, we hypothesize that IL-6 in combination with LDL induces TGF-β1 release from M1 macrophages, which in turn leads to mast cell migration toward macrophages. Adhesion and migration assays as well as ELISA are employed to test this hypothesis.

## Materials and methods

### Cell culture

THP-1 human acute monocytic leukemia cell line was obtained from ATCC (Manassas, Virginia). THP-1 cells were grown in RPMI 1640 (ATCC) medium supplemented with 10% fetal bovine serum (Thermo Fisher Scientific, Waltham, Massachusetts), 1% penicillin/streptomycin (Thermo Fisher), and 0.05mM 2-mercaptoethanol (Millipore-Sigma, Burlington, Massachusetts). The LUVA human mast cell line was a gift from Dr. Steinke (University of Virginia). LUVA cells were grown in SFM medium with StemPro-34 supplement and 2mM L-glutamine (Thermo Fisher). Primary human umbilical vein endothelial cells (HUVEC) were obtained from Thermo Fisher and cultured in Medium 200 supplemented with Low Serum Growth Supplement and Gentamicin/Amphericin B (Thermo Fisher).

### Macrophage differentiation

THP-1 monocytes were differentiated into M0 macrophages by applying 100ng/ml phorbol 12-myristate 13-acetate (PMA) for 48 hours. The polarization of M0 macrophages into M1 or M2 types was achieved by 48-hour culture in the growth medium where PMA was replaced with 20 ng/ml IFN-γ + 100 ng/ml LPS or 20 ng/ml IL-4 + 20 ng/ml IL-13, respectively. M0, M1, and M2 macrophages (commonly denoted as MΦ) were incubated in the medium containing 20ng/ml IL-6, 8 μg/ml LDL, 8 μg/ml oxLDL, or their combination for 24 hours. The medium was then replaced with fresh growth medium, and in next 48 hours, the conditioned medium was collected to generate the following experimental groups: MΦ + LDL, MΦ + OxLDL, MΦ + IL-6, MΦ + LDL + IL-6, and MΦ + OxLDL + IL-6. All the reagents for this study were purchased from Thermo Fisher.

### Enzyme linked immunosorbent assay (ELISA) measurement

TNF-α and TGF-β1 ELISA kits (Eagle Biosciences, Amherst, New Hampshire) were used to measure the TNF-α and TGF-β1 concentration released from macrophages. A 96-well plate was pre-coated with TNF-α or TGF-β1 monoclonal antibody. The macrophage conditioned medium combined with 1N hydrochloric acid (HCl) was placed in a well and stored in 2–8°C for 1 hour to induce TNF-α or TGF-β1 binding to its antibody. HCL was then neutralized by mixing with 1 N sodium hydroxide (NaOH). After the neutralizing procedure, the wells were filled with 100 μL of either ELISA standards or experimental group samples for 1.5 hours at 37°C. These samples were then removed and a 100 μL solution of biotin-conjugate was added to each well and incubated for 1 hour at 37°C. After removing the biotin-conjugate, 100 μL of streptavidin-horse radish peroxidase was added to the well and incubated at 37°C for 30 min. The last two steps were to replace streptavidin-horse radish peroxidase with the substrate solution (15 min incubation) and to replace the substrate solution with the stop solution. The TNF-α and TGF-β1 concentrations were measured from the light absorbance at the wavelength of 450 nm by a microplate reader.

### Static adhesion assay

HUVEC (passage 4–5) were seeded into a 96-well plate and incubated to form an endothelial monolayer. At confluence, the cells were exposed to the conditioned medium from untreated or treated macrophages for 4 hours. This medium was then replaced with RPMI 1640 medium containing THP-1 cells with a concentration of 0.5×10^6^ cells/ml. After a 25-minute incubation, the THP-1 suspension was carefully aspirated, and the wells were washed three times by phosphate buffered saline (PBS) to eliminate non-adherent THP-1 cells. The endothelial monolayer was visualized using a ×10 objective in an inverted microscope (Nikon Eclipse TiS), and images were taken by a digital CCD camera (Qimaging Retiga EXi) at three randomly selected fields. The number of firmly adherent cells were counted.

### μ-slide Chemotaxis assay

LUVA mast cells were cultured in the 30mm petri dish and activated by 100ng/ml PMA for 24 hours, followed by application of cold PBS for cell detachment. Mast cells were then seeded in the center area of the μ-slide (Ibidi USA, Fitchburg, Wisconsin). The growth medium and chemoattractant agent including M0, M1, M2, M1 + LDL, M1 + IL-6, M1 + LDL + IL-6, M1 + OxLDL, M1 + OxLDL + IL-6, or 2ng/ml TGF- β1 were applied to the right-wing part (sink) and left-wing part (source) of the slide, respectively. The trajectories of migrating cells were reconstructed from images taken in the viewing area of the central channel (2mm x 1mm) every 5 minutes for 12 hours. Images were acquired by Basler aca1920 camera connected to the Nikon Ti-S inverted microscope with a ×10 objective.

### Data analysis

Images were analyzed by ImageJ (NIH, Bethesda, Maryland) and “Chemotaxis and migration tools” (v.2, Ibidi). The root-mean-square displacement of the mast cells was calculated from displacements of individual cells, $d_{i}$, i=,…,n, as follows:

1. $$d_{RMS}=\sqrt{\frac{1}{n}\sum_{i=1}^{n}d_{i}^{2}}$$

The directed migration of the cells was assessed by the forward migration index (FMI) calculated as the average ratio of the distance of the cell from its initial location to the end point, $d_{i,c}$, to its path length, $l_{i,c}$:

2. $$FMI=\frac{1}{n}\sum_{i=1}^{n}\;\frac{d_{i,c}}{l_{i,c}}$$

Three to six independent experiments per group were conducted and mean ± SEM was shown in Figures. The statistical significance was determined by one-way ANOVA and Tukey’s post-hoc test in Prism (v. 8, GraphPad Software, San Diego, California).

## Results

### OxLDL induces differentiation of M0 macrophages toward both M1 and M2 types.

**Figure 1:**
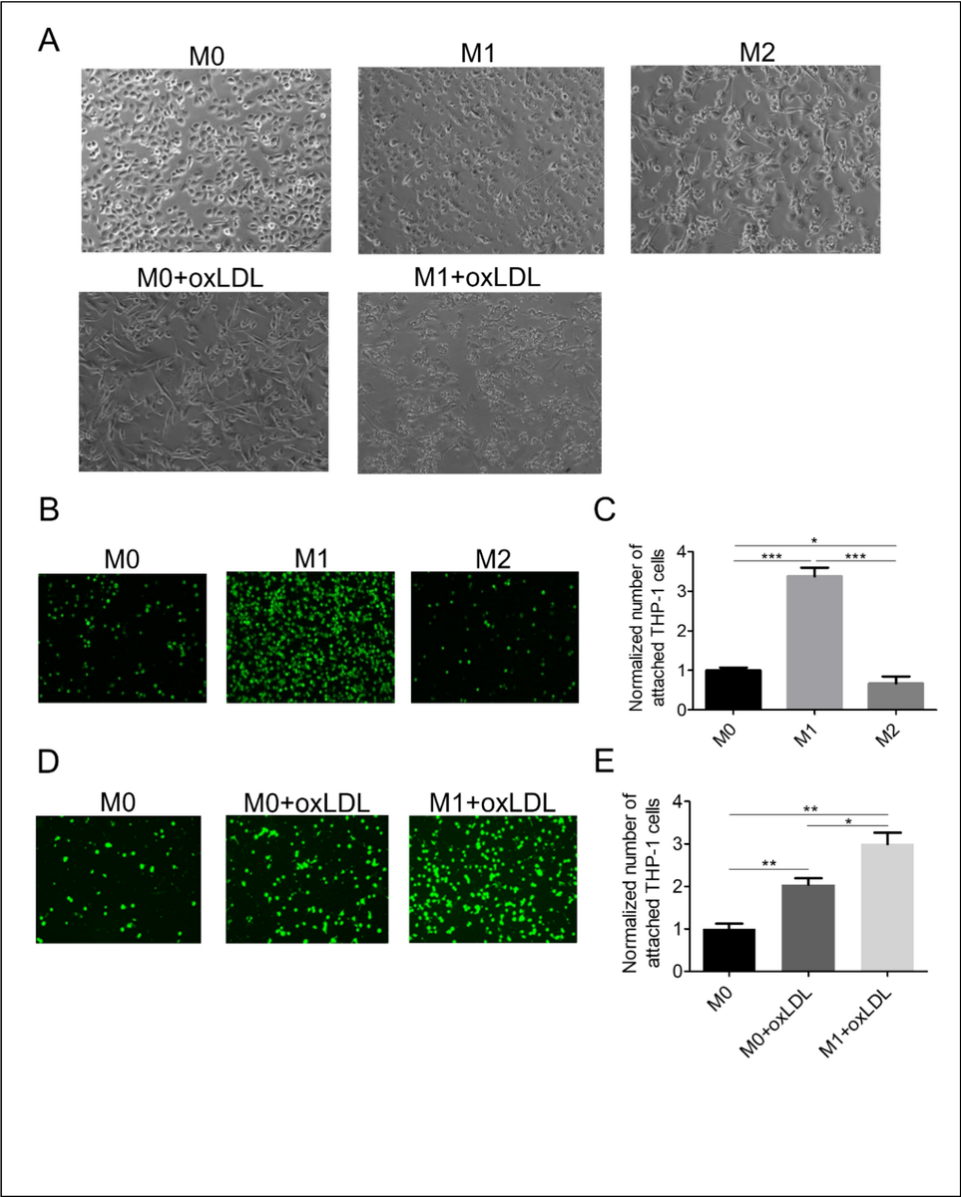
Brightfield images (A) of M0, M1, and M2 macrophages activated or not by oxLDL. Fluorescent images (B, D) and number (C, E) of THP-1 cells attached to HUVEC exposed to conditioned media from respectively untreated and oxLDL-treated M0, M1, and M2 macrophages. . The number of attached cells was normalized to that of the control group. Mean ± SEM of 3 independent experiments. *p<0.05, **p<0.01, ***p<0.001.

**Figure 2:**
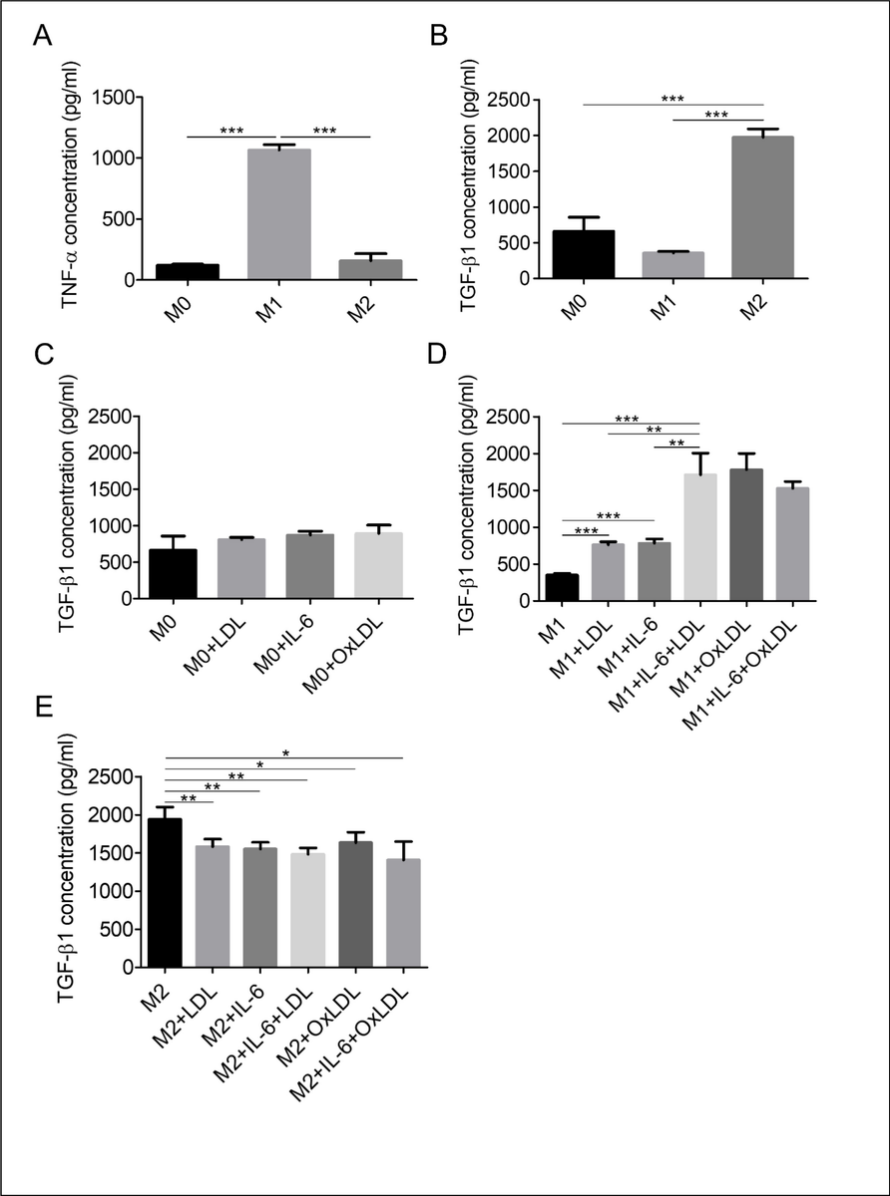
The concentration of TNF-α (A) and TGF-β1 (B) released by M0, M1, and M2 macrophages. (C-E) The concentration of TGF-β1 released by MΦ treated with IL-6, LDL, OxLDL or their combination. Mean ± SEM of 3–5 independent experiments. *p<0.05, **p<0.01, ***p<0.001.

THP-1 cells (Fig. 1A) differentiated into M0 macrophages by PMA treatment are adherent cells with insignificant surface projections (Fig. 1A, see also [27]). Their further differentiation to M1- or M2-types led to uniformly distributed or clustered populations of adherent cells with a large number of surface projections, respectively. The morphology of M0 macrophages treated with oxLDL was similar to that of M1-type. However, some clustering of the cells was also observed, indicating that oxLDL induced heterogeneous differentiation of these cells into M1- and M2-types. This dual transformation of the cells is confirmed by published data and our ELISA measurements, according to which oxLDL-treated M0 macrophages released both TNF-α and TGF-β [13] (Fig. 2A,C). A lot of clustering was observed for M1 macrophages treated with oxLDL (Fig. 1A), which is indicative of M2-type as well as foam cells [28].

Endothelial cells were activated when exposed to the conditioned medium from M1 macrophages but not the medium from M0 or M2 macrophages (Fig. 1B). In particular, monocyte-endothelial adhesion was 3.4 and 5.0 times higher in the M1 macrophage medium than in the M0- or M2-type medium, respectively (p < 0.0001, Fig. 1C). The exposure of M0 macrophages to oxLDL caused a 2-fold increase in monocyte adhesion, which was less than induced by M1 macrophages (Fig. 1D, E). Moreover, there was a decrease in monocyte adhesion between untreated M1 and oxLDL-treated M1 macrophages (3.4 vs 3.0 of M0-type level). This result points out that M1 macrophages partially differentiated into M2 macrophages in the presence of oxLDL.

### OxLDL or LDL combined with IL-6 differentiates M1 macrophages into M2-type

The key phenotypic difference between M1 and M2 macrophages is TNF-α production by and release from the former cells and TGF-β1 production by and release from the latter ones. The concentration of TNF-α in the conditioning medium of M1 macrophages was 1,065 ± 25.51 pg/ml, which was 8.9 and 6.8 times higher than the amount of TNF-α released from M0 (120.0 ± 5.254 pg/ml) and M2 (156.5 ± 29.61 pg/ml) macrophages, respectively (Fig. 2A). All these changes were statistically significant (p < 0.001). The TGF-β1 concentration in the conditioning medium of M2 macrophages (1,750 ± 68.46 pg/ml) was 3.0 and 5.5 times higher than that in the M0 (664.5 ± 111.6 pg/ml) and M1 (357.5 ± 12.72 pg/ml) macrophages, respectively.

When M1 macrophages were exposed to IL-6, LDL, their combination, or oxLDL, they began to release TGF-β1. With their exposure to IL-6 or LDL, the TGF-β1 concentration increased ~ 2.1 fold. It increased by 4.8 and 5.0 times when the cells were treated with IL-6 + LDL and oxLDL, reaching 86% and 90% of the M2-type level, respectively (Fig. 2D). This effect was not observed for M0 or M2 macrophages (Fig. 2C,E). Thus, the majority of M1 macrophages differentiated into M2-type upon exposure to oxLDL or the combination of IL-6 and LDL.

### Mast cells migrate toward macrophages that release high amount of TGF-β1

**Figure 3:**
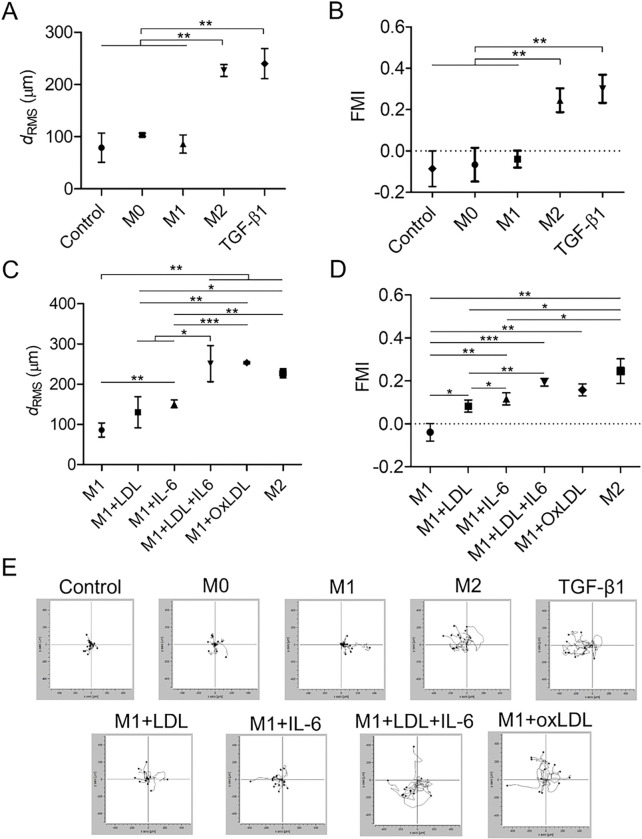
Root mean square displacement (A, C), forward migration index (B, D), and trajectories (E) of mast cells migrating in Ibidi chemotaxis channels for control, M0, M1, M2, M1+IL-6, M1+LDL, M1+LDL+IL-6, M1+OxLDL, and TGF-β1 groups. Mean ± SEM of 3 independent experiments. *p<0.05, **p<0.01, ***p<0.001.

TGF-β1 was shown to induce chemotaxis of mouse mast cells [19], but its effect in human cells was less established. We tested whether TGF-β1 or macrophage conditioning media could be chemotactic for LUVA human mast cells. As seen in Fig. 3A, TGF-β1 significantly increased motility (movement) of LUVA cells, measured by cells’ RMS displacement (239.8 ± 16.59 μm in the TGF-β1 group vs. 78.85 ± 16.19 μm in the control group; p < 0.01). The RMS displacement in the M2 group (226.9 ± 6.59 μm) was slightly less than that in the TGF-β1 group, but it was significantly higher (p < 0.01) than the RMS displacement in the M0 (103.5 ± 1.7 μm) and M1 (85.88 ± 10.09 μm) groups.

The chemotactic migration of LUVA cells was measured by the FMI. This quantity was positive when the cell moved in the direction of a chemotactic source and negative when it moved away. In representative trajectories of LUVA cells (Fig. 3E), the chemotactic source was located on the left of the cells. The highest FMI was for the LUVA cells exposed to TGF-β1 (0.3 ± 0.04). The M2 conditioning medium was insignificantly less chemotactic (0.25 ± 0.03) than TGF-β1. No chemotaxis was observed for the control, M0, and M1 groups, based on negative values of the FMI: −0.08 ± 0.05 (control), −0.07 ± 0.05 (M0), and −0.04 ± 0.02 (M1).

When the LUVA cells were exposed to the conditioning medium of M1 macrophages incubated with IL-6 or LDL, their RMS displacement significantly (p < 0.01) increased to 151.1 ± 9.8 μm and 130 ± 22.29 μm, respectively (Fig. 3C,E). The mast cell activity was further amplified (p < 0.01) with LDL + IL-6 or oxLDL treatment, leading the cell displacements above that in the TGF-β1 group (250.9 ± 25.89μm and 253.2 ± 3.16μm). As evident by the FMI data and cell trajectories (Fig. 3D,E), mast cell migration toward M1 macrophages progressively increased with treatment of the macrophages with LDL alone (0.08 ± 0.02), Il-6 alone (0.11 ± 0.02), oxLDL (0.16 ± 0.02), and LDL + IL-6 (0.2 ± 0.01). All these changes were significantly different (p < 0.05 or less), and the difference between the M1 + LDL + IL-6 and M2 groups were insignificant, indicating that LDL in combination with IL-6 induced complete differentiation of M1 macrophages into M2-type. These data also suggest a key role of IL-6 in mast cell migration toward macrophages.

## Discussion

The results of this study point out that M1 macrophages exposed to oxLDL or a combination of LDL and IL-6 are re-polarized into M2 macrophages. During the repolarization, they release TGF-β1, which activates and attracts the mast cells to the macrophages.

In the clinical context, this study suggests the following mechanism by which LDL and IL-6 induce atherogenesis. First, as a source of cholesterol, native LDL are continuously delivered through blood to tissues in order to regulate membrane fluidity and maintain other functions of tissue-resident cells including macrophages. However, macrophages have a limited capability to phagocyte LDL particles, and when the LDL concentration in blood increases due to genetic reasons or a diet rich in saturated fats, LDL becomes accumulated in the tissues. Some of the excess LDL is modified into oxLDL, which can be phagocyted in large amount by macrophages [29].

Second, when exposed to oxLDL, M1 macrophages release a large amount of pro-inflammatory mediators such as TNF-α [13], which start localized low-grade chronic inflammation. These cells then transform into M2 macrophages and foam cells that release IL-6 and TGF-β1. The tissue concentration of IL-6 also increases during acute inflammation and obesity [30]. Thus, both LDL and IL-6 can be simultaneously present in the intimal layer of the artery.

**Figure 4:**
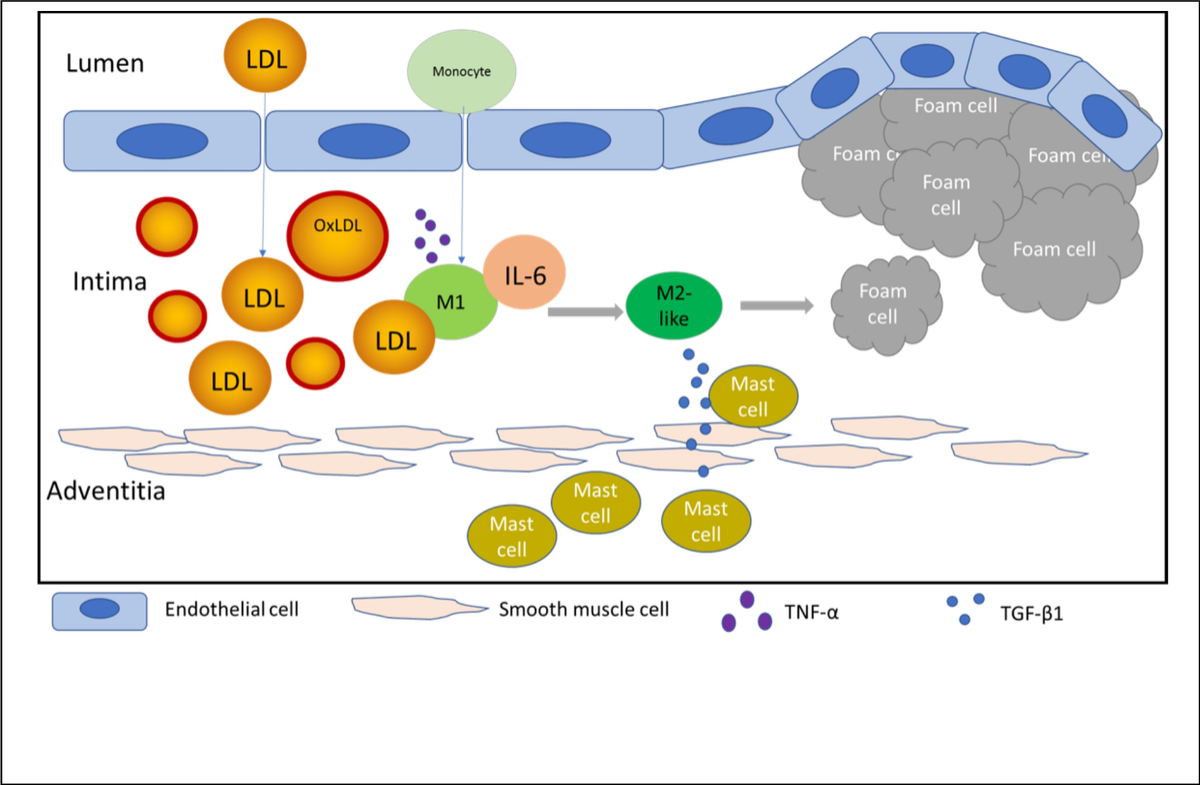
The schematic picture of mast cell migration toward intimal macrophages during atherogenesis.

Third, when combined, these mediators accelerate re-polarization of M1 macrophages to M2 ones, leading to a large concentration of released TGF-β1, which then diffuses to the adventitial layer and interacts with mast cells. According to our study, TGF-β1 causes chemotactic migration of mast cells, particularly, toward the its source, i.e., intimal M2 macrophages or foam cells (Fig. 4). This leads to the co-localization of macrophages and mast cells, evident in later stages of atherosclerosis [16]. The interactions between mast cells and macrophages exacerbates the development of atherosclerosis [13, 31].

To counteract this pathophysiological process, the following therapeutic approaches can be used. The action of IL-6 can be blocked by anti-IL-6R antibody, e.g., tocilizumab or ALX-0061, which effectiveness was proven for autoimmune disease treatment [32, 33]. The mast cell migration can be inhibited by blocking TβR-I and TβR-II, two receptors of TGF-β1 highly expressed in migrating mast cells [21]. The combination of these two therapeutic regimens would prevent inflammatory and wound healing responses in healthy tissues that cause atherogenesis. The effect of pharmacological inhibitors of IL-6 activity and mast cell migration will be tested in our future studies.

## References

[1] Ley K, Laudanna C, Cybulsky MI, Nourshargh S. Getting to the site of inflammation: the leukocyte adhesion cascade updated. Nat Rev Immunol 2007; 7:678–89. 17717539 10.1038/nri2156

[2] van der Wal AC, Das PK, Tigges AJ, Becker AE. Adhesion molecules on the endothelium and mononuclear cells in human atherosclerotic lesions. Am J Pathol 1992; 141:1427–33. 1281621 PMC1886778

[3] Walpola PL, Gotlieb AI, Cybulsky MI, Langille BL. Expression of ICAM-1 and VCAM-1 and monocyte adherence in arteries exposed to altered shear stress. Arterioscler Thromb Vasc Biol 1995; 15:2–10. 7538423 10.1161/01.atv.15.1.2

[4] Weyrich AS, McIntyre TM, McEver RP, Prescott SM, Zimmerman GA. Monocyte tethering by P-selectin regulates monocyte chemotactic protein-1 and tumor necrosis factor-alpha secretion. Signal integration and NF-kappa B translocation. J Clin Invest 1995; 95:2297–303. 7537762 10.1172/JCI117921 PMC295843

[5] Tacke F, Alvarez D, Kaplan TJ, Jakubzick C, Spanbroek R, Llodra J, Monocyte subsets differentially employ CCR2, CCR5, and CX3CR1 to accumulate within atherosclerotic plaques. J Clin Invest 2007; 117:185–94. 17200718 10.1172/JCI28549 PMC1716202

[6] Bobryshev YV. Monocyte recruitment and foam cell formation in atherosclerosis. Micron 2006; 37:208–22. 16360317 10.1016/j.micron.2005.10.007

[7] Tabas I, Bornfeldt KE. Macrophage Phenotype and Function in Different Stages of Atherosclerosis. Circ Res 2016; 118:653–67. 26892964 10.1161/CIRCRESAHA.115.306256 PMC4762068

[8] Frostegard J, Ulfgren AK, Nyberg P, Hedin U, Swedenborg J, Andersson U, Cytokine expression in advanced human atherosclerotic plaques: dominance of pro-inflammatory (Th1) and macrophage-stimulating cytokines. Atherosclerosis 1999; 145:33–43. 10428293 10.1016/s0021-9150(99)00011-8

[9] Barros MH, Hauck F, Dreyer JH, Kempkes B, Niedobitek G. Macrophage polarisation: an immunohistochemical approach for identifying M1 and M2 macrophages. PLoS One 2013; 8:e80908. 24260507 10.1371/journal.pone.0080908 PMC3829941

[10] Glaros T, Larsen M, Li L. Macrophages and fibroblasts during inflammation, tissue damage and organ injury. Front Biosci (Landmark Ed) 2009; 14:3988–93. 19273328 10.2741/3506

[11] Wang C, Chen J, Sun L, Liu Y. TGF-beta signaling-dependent alleviation of dextran sulfate sodium-induced colitis by mesenchymal stem cell transplantation. Mol Biol Rep 2014; 41:4977–83. 24737572 10.1007/s11033-014-3364-6

[12] Oh J, Riek AE, Weng S, Petty M, Kim D, Colonna M, Endoplasmic reticulum stress controls M2 macrophage differentiation and foam cell formation. J Biol Chem 2012; 287:11629–41. 22356914 10.1074/jbc.M111.338673 PMC3320912

[13] Chen C, Khismatullin DB. Oxidized low-density lipoprotein contributes to atherogenesis via co-activation of macrophages and mast cells. PLoS One 2015; 10:e0123088. 25811595 10.1371/journal.pone.0123088 PMC4374860

[14] Badrnya S, Schrottmaier WC, Kral JB, Yaiw KC, Volf I, Schabbauer G, Platelets mediate oxidized low-density lipoprotein-induced monocyte extravasation and foam cell formation. Arterioscler Thromb Vasc Biol 2014; 34:571–80. 24371083 10.1161/ATVBAHA.113.302919

[15] Lindstedt KA, Mayranpaa MI, Kovanen PT. Mast cells in vulnerable atherosclerotic plaques--a view to a kill. J Cell Mol Med 2007; 11:739–58. 17760836 10.1111/j.1582-4934.2007.00052.x PMC3823253

[16] Xu JM, Shi GP. Emerging role of mast cells and macrophages in cardiovascular and metabolic diseases. Endocr Rev 2012; 33:71–108. 22240242 10.1210/er.2011-0013 PMC3365842

[17] Wezel A, Quax PH, Kuiper J, Bot I. The role of mast cells in atherosclerosis. Hamostaseologie 2015; 35:113–20. 25377048 10.5482/HAMO-14-08-0034

[18] Kaartinen M, Penttila A, Kovanen PT. Accumulation of activated mast cells in the shoulder region of human coronary atheroma, the predilection site of atheromatous rupture. Circulation 1994; 90:1669–78. 7923651 10.1161/01.cir.90.4.1669

[19] Gruber BL, Marchese MJ, Kew RR. Transforming growth factor-beta 1 mediates mast cell chemotaxis. J Immunol 1994; 152:5860–7. 7515916

[20] Halova I, Draberova L, Draber P. Mast cell chemotaxis - chemoattractants and signaling pathways. Front Immunol 2012; 3:119. 22654878 10.3389/fimmu.2012.00119 PMC3360162

[21] Olsson N, Piek E, ten Dijke P, Nilsson G. Human mast cell migration in response to members of the transforming growth factor-beta family. J Leukoc Biol 2000; 67:350–6. 10733095 10.1002/jlb.67.3.350

[22] Fernando MR, Reyes JL, Iannuzzi J, Leung G, McKay DM. The pro-inflammatory cytokine, interleukin-6, enhances the polarization of alternatively activated macrophages. PLoS One 2014; 9:e94188. 24736635 10.1371/journal.pone.0094188 PMC3988054

[23] Escate R, Padro T, Badimon L. LDL accelerates monocyte to macrophage differentiation: Effects on adhesion and anoikis. Atherosclerosis 2016; 246:177–86. 26800307 10.1016/j.atherosclerosis.2016.01.002

[24] Shashkin P, Dragulev B, Ley K. Macrophage differentiation to foam cells. Curr Pharm Des 2005; 11:3061–72. 16178764 10.2174/1381612054865064

[25] E L, Fragakis N, Ioannidou E, Bounda A, Theodoridou S, Klonizakis P, Increased levels of proinflammatory cytokines in children with family history of coronary artery disease. Clin Cardiol 2010; 33:E6–10. 10.1002/clc.20434 PMC6653360 20229495

[26] Meisinger C, Baumert J, Khuseyinova N, Loewel H, Koenig W. Plasma oxidized low-density lipoprotein, a strong predictor for acute coronary heart disease events in apparently healthy, middle-aged men from the general population. Circulation 2005; 112:651–7. 16043640 10.1161/CIRCULATIONAHA.104.529297

[27] Stewart DA, Yang Y, Makowski L, Troester MA. Basal-like breast cancer cells induce phenotypic and genomic changes in macrophages. Mol Cancer Res 2012; 10:727–38. 22532586 10.1158/1541-7786.MCR-11-0604 PMC3640417

[28] Rios de la Rosa JM, Tirella A, Gennari A, Stratford IJ, Tirelli N. The CD44-Mediated Uptake of Hyaluronic Acid-Based Carriers in Macrophages. Adv Healthc Mater 2017; 6. 10.1002/adhm.201601012 27990775

[29] Brown MS, Goldstein JL. Lipoprotein metabolism in the macrophage: implications for cholesterol deposition in atherosclerosis. Annu Rev Biochem 1983; 52:223–61. 6311077 10.1146/annurev.bi.52.070183.001255

[30] Kern L, Mittenbuhler MJ, Vesting AJ, Ostermann AL, Wunderlich CM, Wunderlich FT. Obesity-Induced TNFalpha and IL-6 Signaling: The Missing Link between Obesity and Inflammation-Driven Liver and Colorectal Cancers. Cancers (Basel) 2018; 11. 10.3390/cancers11010024 PMC6356226 30591653

[31] Bot I, Shi GP, Kovanen PT. Mast cells as effectors in atherosclerosis. Arterioscler Thromb Vasc Biol 2015; 35:265–71. 25104798 10.1161/ATVBAHA.114.303570 PMC4304944

[32] Ho LJ, Luo SF, Lai JH. Biological effects of interleukin-6: Clinical applications in autoimmune diseases and cancers. Biochem Pharmacol 2015; 97:16–26. 26080005 10.1016/j.bcp.2015.06.009

[33] Mihara M, Moriya Y, Kishimoto T, Ohsugi Y. Interleukin-6 (IL-6) induces the proliferation of synovial fibroblastic cells in the presence of soluble IL-6 receptor. Br J Rheumatol 1995; 34:321–5. 7788145 10.1093/rheumatology/34.4.321

